# Organic selenium supplementation increased selenium concentrations in ewe and newborn lamb blood and in slaughter lamb meat compared to inorganic selenium supplementation

**DOI:** 10.1186/1751-0147-50-7

**Published:** 2008-03-31

**Authors:** Arvid Steen, Turid Strøm, Aksel Bernhoft

**Affiliations:** 1 Department of Production Animal Clinical Sciences, Norwegian School of Veterinary Science, P.O. Box 8146 Dep., NO-0033 Oslo, Norway; 2 Bioforsk Organic Food and Farming Division, Tingvoll gard, NO-6630 Tingvoll, Norway; 3 Department of Toxicology, National Veterinary Institute, Box 8156 Dep., NO-0033 Oslo, Norway

## Abstract

**Background:**

Selenium is part of the antioxidant defence system in animals and humans. The available selenium concentration in soil is low in many regions of the world. The purpose of this study was to evaluate the effect of organic versus inorganic selenium supplementation on selenium status of ewes, their lambs, and slaughter lambs.

**Methods:**

Ewes on four organic farms were allocated five or six to 18 pens. The ewes were given either 20 mg/kg inorganic selenium as sodium selenite or organic selenium as selenized nonviable yeast supplementation for the two last months of pregnancy. Stipulated selenium concentrations in the rations were below 0.40 mg/kg dry matter. In addition 20 male lambs were given supplements from November until they were slaughtered in March. Silage, hay, concentrates, and individual ewe blood samples were taken before and after the mineral supplementation period, and blood samples were taken from the newborn lambs. Blood samples from ewes and lambs in the same pens were pooled. Muscle samples were taken from slaughter lambs in March. Selenium concentrations were determined by atomic absorption spectrometry with a hydride generator system. In the ANOVA model, selenium concentration was the continuous response variable, and selenium source and farm were the nominal effect variables. Two-sample *t*-test was used to compare selenium concentrations in muscle samples from the slaughtered lambs that received either organic or inorganic selenium supplements.

**Results:**

In all ewe pens the whole blood selenium concentrations increased during the experimental period. In addition, ewe pens that received organic selenium had significantly higher whole blood selenium concentrations (mean 0.28 μg/g) than ewe pens that received inorganic selenium (mean 0.24 μg/g). Most prominent, however, was the difference in their lambs; whole blood mean selenium concentration in lambs from mothers that received organic selenium (mean 0.27 μg/g) was 30% higher than in lambs from mothers that received inorganic selenium (mean 0.21 μg/g). Slaughter lambs that received organic selenium had 50% higher meat selenium concentrations (mean 0.12 mg/kg wet weight) than lambs that received inorganic selenium (mean 0.08 mg/kg wet weight).

**Conclusion:**

Organic selenium supplementation gave higher selenium concentration in ewe and newborn lamb blood and slaughter lamb meat than inorganic selenium supplementation.

## Background

Selenium is part of the antioxidant defence system in animals and humans. The selenium concentration in soil is low in many parts of the world including the Nordic countries where it is poorly available because of low soil pH [1-5]. Animals fed roughage grown in selenium-deficient areas and not supplemented with minerals are vulnerable to oxidant stress. Clinical consequences include ill thrift, reproductive problems, lowered resistance to infectious diseases such as mastitis, and nutritional myopathies [6]. Clinical selenium deficiency is rare among humans. In certain areas of China, selenium deficiency predisposes patients to Keshan disease, an endemic viral cardiomyopathy which primarily affects children and young women. In Siberian Russia and China, growing children with selenium deficiency may develop chronic osteoarthropathy (Kashin-Beck disease) [7]. However, less-overt selenium deficiencies are probably of more significance. Selenium is required for the proper functioning of the immune system, and appears to be a key nutrient in counteracting the development of virulence and inhibiting HIV progression to AIDS [8]. According to experimental animal studies and some observational epidemiological studies in humans, higher selenium intakes might reduce the risk of certain types of cancer [9,10].

Cereal products and vegetables grown in the Nordic countries, with the exception of Finland, have low selenium content. This contrasts with wheat from North America which has high selenium content and is frequently imported into Norway and Iceland. Feeds to animals in the Nordic countries need to be supplemented with selenium to avoid deficiencies [1,11,12]. Selenium intake among humans in Sweden and Denmark is below Nordic Nutrition Recommendations 2004 [13]. In Norway and Iceland, the selenium intake in humans has been sufficient because of high-selenium wheat imported from North America. This situation could change in Norway because of more home-grown wheat production in recent years.

Pehrson [14] concludes that supplementation of farm animal diets with organic selenium instead of inorganic selenium will increase selenium intake in animals and non-vegetarian humans. The purpose of this investigation was to evaluate the effect of organic versus inorganic selenium supplementation on selenium status in ewe and newborn lamb blood and slaughter lamb meat.

## Methods

### Farms, Animals, Feeding, and Samples

Sheep from two inland and two coastal organic farms were selected for the trial. On each farm, ewes were randomly allocated into pens of five or six. Each pen received an organically approved mineral supplement containing either 20 mg/kg inorganic selenium as sodium selenite (9 pens), or organic selenium (9 pens). The organic selenium ingredient, mostly selenomethionine, was selenized nonviable yeast produced by *Saccharomyces cereviciae *[15]. The mineral supplements were mixed in a commercial mill.

The sheep were fed a ration which contained grass silage, hay and concentrates. The ewes were given 0.3 to 1.0 kg of concentrates before lambing depending on the number of lambs they were carrying. Grass silage and hay were fed ad libitum. The ewes in each pen consumed 20 g per head of mineral supplement each day on average during the experimental period, which was the last two months before lambing.

The selenium concentrations in silage and hay from all four farms were below 0.05 mg/kg dry matter. Selenium concentrations in the two farms that were located at the coast of Norway were higher (0.03 to 0.05 mg/kg dry matter) than in the two farms located in the inland in Eastern Norway (<0.01 to 0.02 mg/kg dry matter). The selenium concentrations in the roughage were not different from the beginning to the end of the experimental period. Selenium concentrations in concentrates were from 0.17 to 0.57 mg/kg dry matter. Assuming a total dry matter intake of between 1.6 to 1.8 kg per pregnant ewe per day [16], stipulated selenium concentrations in the rations without mineral supplements were between 0.06 to 0.15 mg/kg dry matter; with mineral supplements the total ration selenium concentrations were below 0.40 mg/kg dry matter.

In addition, 20 male lambs on one farm that were too small to be slaughtered in the autumn were given 0.15 kg molasses ensiled barley that was threshed early (50 to 65% dry matter), 0.15 kg commercial concentrates, grass silage ad libitum and free access to mineral supplements up to an average of 20 g per head from November until they were slaughtered in March. The male lambs were randomly allocated to organic selenium supplement (13 lambs) or inorganic selenium supplement (7 lambs) irrespective of the supplementation their mother's had received six months previously. The cause of the skewed distribution between treatments was that the host farmer only had one large and one small pen available for the experiment.

The selenium concentrations in four samples of both the organic selenium and inorganic selenium mineral mixtures were analyzed before being sent to the farmers, and mean concentrations were 19.9 and 23.0 mg selenium/kg, respectively. Silage, hay, concentrates, and individual ewe blood samples were taken just before the mineral supplementation period, and after lambing. Blood samples from ewes in the same pens were pooled before analysis. Blood samples from lambs from mothers in the same pens were taken within the first week post partum and were also pooled before analysis. Blood samples were drawn from the jugular vein (Venoject II, with lithium heparin). The whole blood samples were posted the same day, frozen at arrival the day after and stored until analysis of all samples. Muscle samples from winter-slaughtered male lambs were taken caudoproximally to the carpus, *musculus flexor carpi ulnaris*.

There are no ethical implications as both groups got selenium supplements.

### Analyses

Silage, hay, concentrate, blood and muscle samples were analysed for selenium at the National Veterinary Institute, Norway. Selenium concentrations were determined by atomic absorption spectrometry with a hydride generator system [17], using a Varian SpectrAA-30 with a VGA-76 vapour generation accessory. Before analysis, each sample was prepared by oxidative digestion in a mixed solution with concentrated nitric and perchloric acids, using an automated system with a Tecator 1012 Controller and 1016 Digester heating unit. This method is accredited (NS-EN ISO/IEC 170225). All selenium concentrations were calculated as μg per g blood or feed dry matter; 1 μg/g blood corresponds to 1.052 μg/ml [18]. The detection limit was 0.01 μg/g.

### Statistics

Selenium concentrations in pooled samples from ewes and newborn lambs were compared between pens receiving mineral supplement with either organic or inorganic selenium using the FIT MODEL platform in JMP (JMP Version 6, SAS Institute Inc., Cary, NC). In the ANOVA-model, selenium was treated as a continuous response variable; and selenium source and farm were nominal (discrete) effect variables. The model was also tested for interaction between selenium source and farm. Two-sample *t*-test was used to compare selenium concentrations in muscle samples from male lambs that received either organic or inorganic selenium supplements. Differences were declared at the 5% level.

## Results

**Figure 1:**
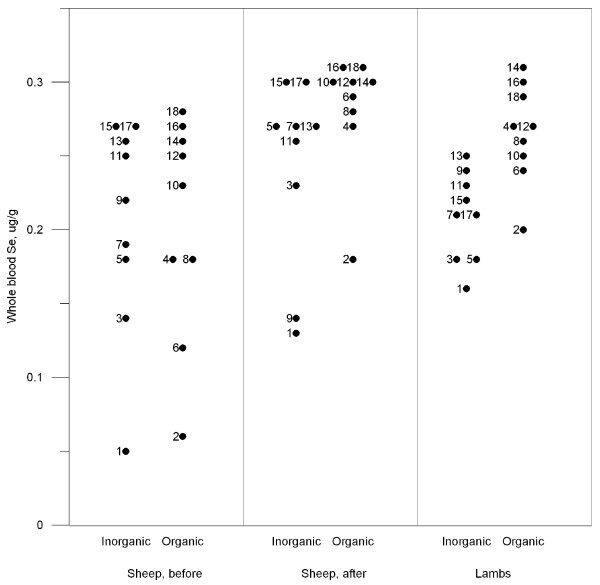
**Whole blood selenium concentrations in ewes before supplementation, and in ewes and their newborn lambs after inorganic or organic selenium supplementation the last two month of pregnancy**. The numbers 1 to 18 corresponds to the pooled blood samples from ewes and lambs in the same pens before and after the supplementation period.

Whole blood selenium concentrations from the sheep in all four farms are plotted in figure 1. There were no differences between pens within farm before the experimental period (mean 0.20 μg/g). After two months with mineral supplements, both groups of ewes had in general higher whole blood selenium concentrations compared to before the experimental period. In addition, ewes that received organic selenium had significantly higher whole blood selenium concentrations (0.28 ± 0.01 μg/g; mean ± SEM) than ewes that received inorganic selenium (0.24 ± 0.02 μg/g). Most prominent, however, was the significant difference in the lambs; lambs from mothers that received organic selenium had nearly 30% higher whole blood selenium concentrations (0.27 ± 0.01 μg/g) than lambs from mothers that received inorganic selenium (0.21 ± 0.01 μg/g).

**Figure 2:**
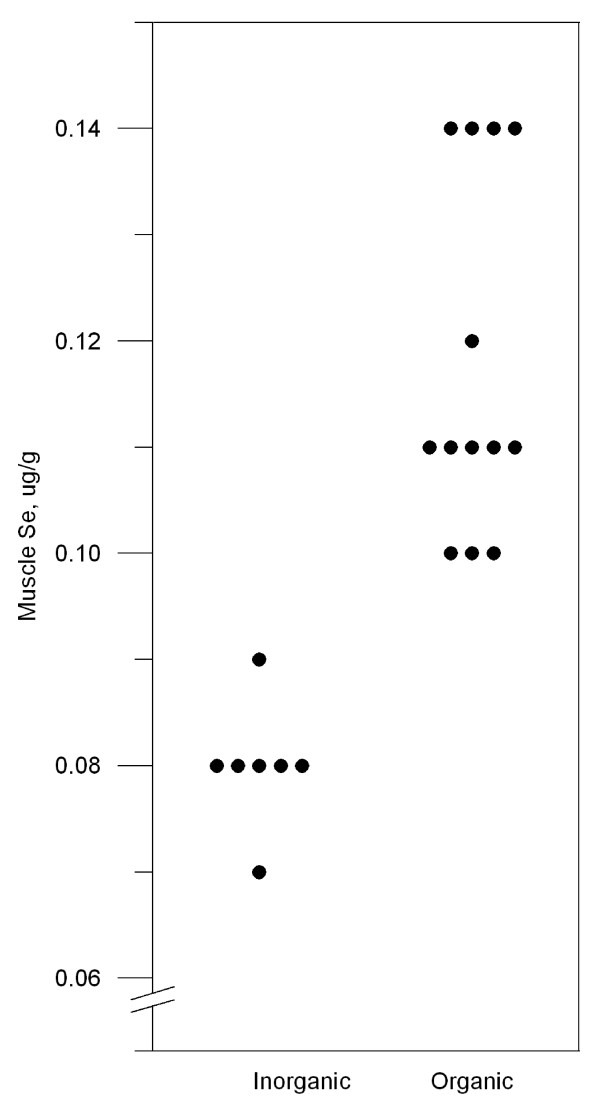
Meat selenium concentrations in slaughter lambs after four months of inorganic or organic selenium supplementation.

Muscle selenium concentrations from 7 lambs that received inorganic selenium supplement and 13 lambs that received organic selenium are plotted in figure 2. Slaughter lambs that received organic selenium had significantly higher meat selenium concentrations (mean 0.12 mg/kg wet weight) than lambs that received inorganic selenium (mean 0.08 mg/kg wet weight).

## Discussion

Organic selenium supplementation gave higher selenium concentrations than inorganic selenium in ewe whole blood (figure 1). This is in accordance with some trials with cattle [19,20] and pigs [21], although more equivocal results have been obtained in pigs [22,23].

Organic selenium supplementation gave also higher selenium concentrations than inorganic selenium in whole blood of newborn lambs. This is comparable with trials in cattle [24] and pigs [25] that measured selenium concentrations in offspring from mothers fed organic selenium compared to offspring from mothers receiving inorganic selenium. In the cattle trial, however, the calves were sampled after three weeks; the newborn lambs in our experiment were sampled within the first week postpartum.

Organic selenium supplementation gave higher selenium concentrations than inorganic selenium in lamb meat. This increased selenium concentration was highly significant even in this small trial with only 13 plus 7 animals in the organic and inorganic groups respectively. The higher concentration in meat is in accordance with trials that compared organic and inorganic selenium sources in other species; organic selenium was superior to inorganic selenium in increasing the selenium content in meat of cattle [19,26] and swine [27,28]. The male lambs were randomly allocated to organic or inorganic selenium supplement irrespective of what their mothers had received before lambing six months ago. Because the lambs and their mothers did not get any mineral supplements during the six months on pasture, we assume that there was no carry-over effect from the two different selenium sources.

Only two pens with ewes had marginally deficient whole blood selenium concentrations before the supplemental period started (figure 1, pen 1 and 2). There are different definitions of marginal deficiencies in the literature and we chose to use the definition from The National Veterinary Institute in Norway which defines whole blood selenium concentrations between 0.05 to 0.10 μg/g to be marginal. All four farms had participated in an earlier screening of mineral content [1]. Thus, the farmers were aware of their low selenium concentrations in soil and roughage and had given their sheep selenium-enriched concentrates and different mineral mixtures, but apparently in varying amounts before the trial started.

## Conclusion

Ewes that received organic selenium had significantly higher whole blood selenium concentrations than ewes that received inorganic selenium. Lambs from mothers that received organic selenium had nearly 30% higher whole blood selenium concentrations than lambs from mothers that received inorganic selenium. Organic selenium supplementation gave 50% higher selenium concentration in lamb meat than inorganic selenium supplementations. Our findings support Pehrson's conclusion [14] that supplementation of farm animal diets with organic selenium instead of inorganic selenium will increase selenium status in ewes, newborn lambs and slaughter lambs.

## Competing interests

The author(s) declare that they have no competing interests.

## Authors' contributions

AS was responsible for blood sampling, coordination of the study, performed the statistical analysis and drafted the manuscript. TS was responsible for feed sampling and coordination of the study. AB was responsible for the selenium analyses. All authors participated in the design of the study, helped to draft the manuscript, and read and approved the final manuscript.
